# Mr. T. F. Gardner

**Published:** 1912-08-10

**Authors:** 


### Obituary.

Mr. T. F. Gardner, M.D., died at his home in Bournemouth on August 1. He graduated at Durham University in 1902, having taken his M.R.C.P. three years previously. A University College Hospital man, his appointments included those of physician to the Royal Victoria and West Hants Hospital and previously surgeon to the Cottage Hospital, Bournemouth.

